# Health-enhancing physical activity induce beneficial skeletal muscle mitochondrial adaptations in individuals with rheumatoid arthritis

**DOI:** 10.1007/s10067-025-07734-z

**Published:** 2025-10-20

**Authors:** Emily Shorter, Elena Ossipova, Estela Santos Alves, Helena Idborg, Jone Vanluyten, Eva Kosek, Liu Zhengye, Birgitta Nordgren, Cecilia Fridén, Ferdinand von Walden, Christer Malm, Per-Johan Jakobsson, Christina H. Opava, Marina Korotkova, Ingrid E. Lundberg, Johanna T. Lanner

**Affiliations:** 1 Department of Physiology and Pharmacology, Molecular Muscle Physiology and Pathophysiology Group, Karolinska Institutet, Solnavagen 9, 17177 Stockholm, Sweden; 2 Department of Medicine, Division of Rheumatology, Karolinska Institutet, SE-17176 Stockholm, Sweden; 3 Karolinska University Hospital, Stockholm, Sweden; 4 Department of Clinical Neuroscience, Karolinska Institutet, Osher Centrum, 17177 Stockholm, Sweden; 5 Department of Neurobiology, Care Sciences and Society, Division of Physiotherapy, Karolinska Institutet, 14183 Huddinge, Sweden; 6 Department of Women and Children’s Health, Division of Pediatric Neurology, Karolinska Institutet, 17176 Stockholm, Sweden; 7 Department of Community Medicine and Rehabilitation, Section of Sports Medicine, Umeå University, 90197 Umeå, Sweden; 8 Department of Surgical Sciences, Uppsala University, Uppsala Academic Hospital, 751 85 Uppsala, Sweden; 9 Medical Unit Occupational Therapy and Physiotherapy, Women’s Health and Allied Health Professionals, Karolinska University Hospital, 141 86 Stockholm, Sweden; 10 Department of Healthcare and Welfare, Division of Physiotherapy, Mälardalen University, 721 23 Västerås, Sweden

**Keywords:** Exercise adaptation, Health-enhancing physical activity, Mitochondria, Muscle biopsy, Rheumatoid arthritis, Skeletal muscle

## Abstract

**Objective:**

Rheumatoid arthritis (RA) is a common chronic systemic inflammatory disease that causes musculoskeletal impairments and fatigue. Physical activity is recommended for individuals with RA, and health-enhancing physical activity (HEPA) has been shown to improve health perception and physical fitness in this group. However, the molecular adaptations of skeletal muscle in response to an exercise intervention are still unexplored in individuals with RA. This study aimed to assess the skeletal muscle response to a 2-year HEPA intervention in individuals with RA.

**Methods:**

Thirteen individuals with RA (65 ± 2 years old, 13 ± 2 years disease duration) participated. The 2-year HEPA intervention involved 150 min of weekly moderately intense aerobic activity and twice-weekly circuit training. Practical and theoretical physiotherapist support was available the first year, but not the second year. Skeletal muscle biopsies, functional assessments, and mass spectrometry-based proteomics analysis were conducted.

**Results:**

Compliance was high the first year but dropped significantly the second year. Functional improvements in strength, endurance, and lower extremity muscle function (TST) were observed after year 1. Proteomics analysis revealed significant enrichment of mitochondrial proteins including COX8A, citrate synthase, M2OM, NDUFA6, NDUFS2, and VDAC3 after year 1, indicating positive muscle adaptations. However, these changes regressed to baseline levels by year 2.

**Conclusion:**

HEPA can induce beneficial mitochondrial adaptations in skeletal muscle of individuals with RA. However, insufficient compliance and progression in HEPA exercise load led to a reversal of these adaptations. Continuous support and motivation are crucial for maintaining and progressing exercise levels and muscle health in individuals with RA.

**Table:** 

**Key points** • *Health-enhancing physical activity (HEPA) can induce beneficial mitochondrial adaptations in the skeletal muscle proteome of individuals with RA*. • *Positive effects on mitochondrial protein levels aligned with the participants compliance to the HEPA intervention*. • *Results emphasizes that sustaining and progressing exercise regimen is crucial to maintain beneficial adaptations for individuals with RA*.

**Supplementary Information:**

The online version contains supplementary material available at 10.1007/s10067-025-07734-z.

## Introduction

Rheumatoid arthritis (RA) is a common chronic, systemic inflammatory disease that primarily affects synovial joints [1] but is also associated with musculoskeletal impairments and fatigue [2–4]. Physical activity and exercise are recommended for people with RA to prevent or reduce disability [5]. Aerobic and resistance training interventions have been shown to lower RA-associated cardiovascular risk factors, such as blood triglycerides and high-density lipoprotein levels, while reducing body fat, improving aerobic capacity (VO2 max), and enhancing health assessment questionnaire scores [6, 7]. Notably, exercise influences molecular pathways involved in inflammation, capillary growth, and skeletal muscle remodeling, as evidenced in related conditions like polymyositis and dermatomyositis [8].

Despite these benefits, adherence to regular exercise remains low among individuals with RA, with only 10–25% engaging in routine physical activity [9]. To address this, a 2-year intervention based on WHO guidelines for health-enhancing physical activity (HEPA) was designed for individuals with RA [10, 11]. HEPA includes 150 min of weekly moderately intense aerobic physical activity and twice-weekly muscle strength training to improve health perception and physical fitness [11].

In this HEPA intervention, 220 individuals participated [9, 11], with output measures focusing on general health perception, physical fitness, and pain and fatigue. Over the first year, physiotherapist support helped 75% of participants adhere to HEPA, leading to improvements in health perception and muscle endurance. However, adherence dropped to 27% in the second year, following the withdrawal of professional support, and no further improvements were observed [9].

Although HEPA and similar interventions have been shown to contribute to improvement in health perception, physical fitness [9], and cardiometabolic health in individuals with RA [6, 7], the skeletal muscle response remains unclear. Individuals with RA are known to exhibit muscle weakness and poorer muscle quality [2, 3], which impede their daily activities as well as their ability to work [12]. Skeletal muscle, which constitutes about 50% of body mass, is an energy-demanding tissue essential for movement and respiration. Muscle contraction and maintenance depend on energy-intensive processes, primarily powered by oxidative phosphorylation (OXPHOS) in the mitochondria. This process produces adenosine triphosphate (ATP) by moving electrons through the electron transport chain and creating a proton gradient used by ATP synthase. Hence, functioning mitochondria are required to maintain normal muscle function.

To evaluate skeletal muscle responses to HEPA, we conducted functional tests and collected vastus lateralis biopsies before the intervention and after 1 and 2 years of HEPA. Mass spectrometry-based proteomics was used to analyze these biopsies, providing insight into molecular mechanisms underlying the observed benefits of exercise in RA.

## Methods

### Participants and sampling

This is an exploratory sub-study of the HEPA intervention study focusing on the effects of HEPA intervention on repeat muscle biopsies (REF). Enrolled participants were 65 ± 2 years old white female subjects with a mean disease duration of 13 ± 2 years and an average disease activity score (DAS28) of 1.88 ± 0.42 (*N* = 13). At inclusion, enrolled participants had no previous experience of HEPA and exhibited no major comorbidities preventing them from being physically active. Median activity limitation at the start of the intervention was 0.25, with 3 being the maximum limitation according to the Health Assessment Questionnaire Disability Index, HAQ-DI [13]. Detailed participant information is displayed in Supplemental Table 1. In this HEPA sub-study, muscle biopsies from before starting the exercise program and after 1 and 2 years of follow-up were investigated. Only participants (*n* = 6) with all three biopsy samples were included (*n* = 18 biopsies). Muscle biopsies from m. vastus lateralis were collected under local anesthesia using the conchotome technique [14] at Karolinska University Hospital and Sunderbyn Hospital in Sweden. Stockholm regional research ethics committee approved the study (2010/1232–31/1). The six patients whose muscle biopsies were used for proteomic analyses were all on disease-modifying antirheumatic drugs (DMARDs), but none was using glucocorticoids. This is an important consideration, as glucocorticoids are known to induce catabolic responses in skeletal muscle [15] which could potentially interfere with exercise adaptation. Biopsies were snap-frozen in liquid nitrogen and stored at − 80 °C.

### The HEPA intervention

**Fig. 1:**
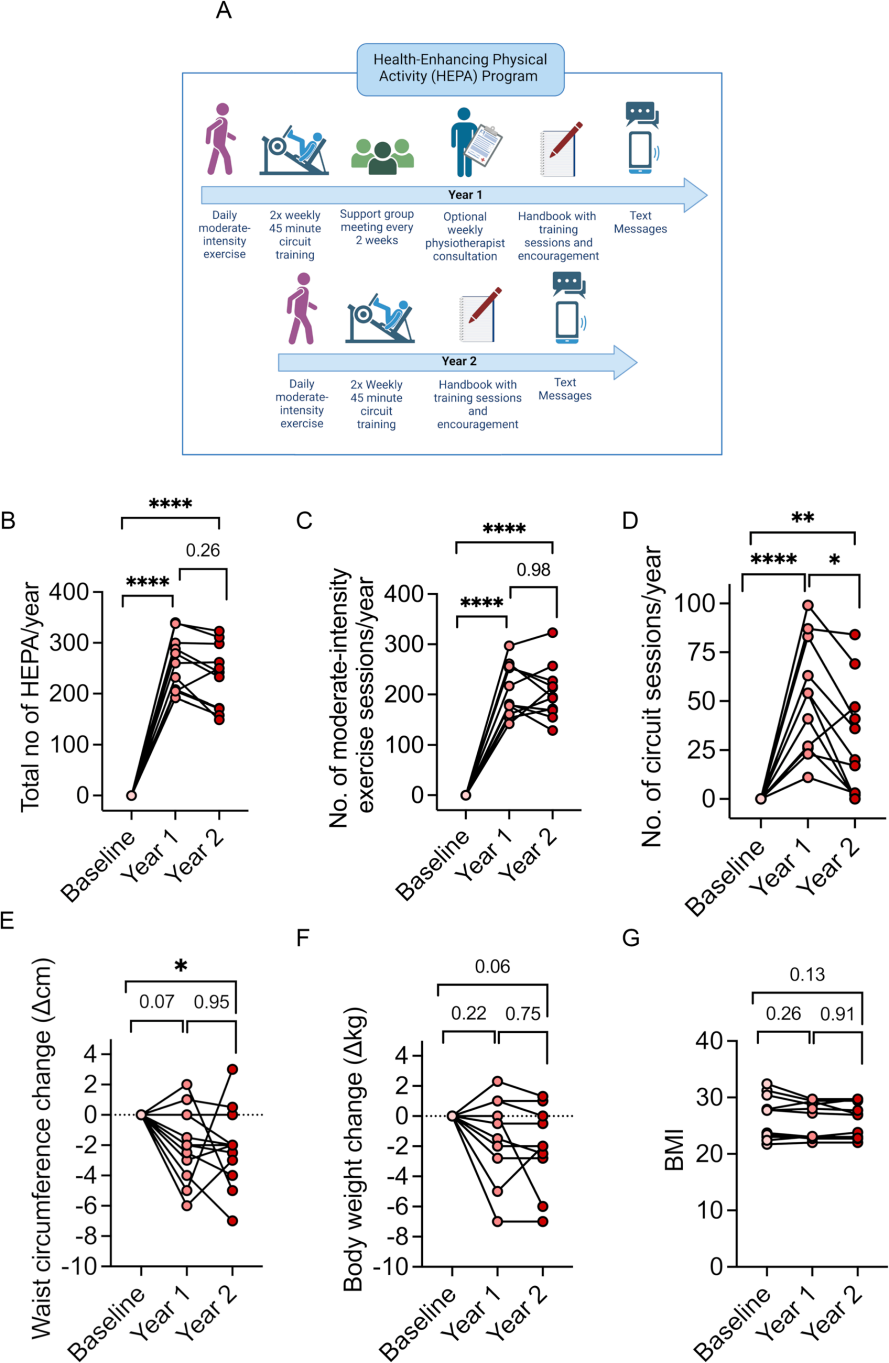
The health-enhancing physical activity (HEPA) program and compliance. **A** Schematic overview of the weekly activities required of the health-enhancing physical activity (HEPA) intervention over the 2 years of the study. At the start of the intervention, physiotherapists specializing in rheumatology tailored training programs to each participant’s abilities. The exercise consisted of daily moderate-intensity physical activity (> 30 min) together with twice-weekly circuit training, and participants received weekly text messages to monitor and encourage HEPA and for self-reporting of the performed sessions. During year 1, there were 1-h weekly consultations from physiotherapists as well as 1-h biweekly support groups. During year 2, the participants maintained the individualized HEPA program, with optional self-organized support group meetings without physiotherapist support. Created in https://BioRender.com. **B**–**D** Adherence to the intervention, the total number of HEPA per year (**B**), total number of moderate-intensity exercise sessions per year (**C**), and **D** total number of circuit sessions per year attended by participants. **E**–**G** Anthropometric changes over the 2-year HEPA intervention with change in **E** waist circumference, **F** body weight, and **G** body mass index (BMI). *p < 0.05, **p < 0.01, **** *p* < 0.0001, repeated measures ANOVA

This exercise intervention aimed to have participants initiate and maintain HEPA for 2 years, following WHO guidelines [10]. Each year, participants were to engage in moderate-intensity physical activity (> 30 min) 5 days a week along with twice-weekly circuit training and self-reporting by text messages. The circuit training, of 45 min, included a warm-up, five strengthening exercises of major skeletal muscle groups, aerobic exercises, and stretching. Strength exercises involved three laps and ten repetitions per exercise and lap (3 × 10 repetitions) at 50–80% of one repetition maximum (1 RM). Hydraulic exercise machines (Easyline by Technogym) ensured adjustment of the concentric resistance to the performance speed. Aerobic exercises were performed in 30-s intervals at 60–80% of maximum heart rate, with three laps of ten exercises each. Importantly, the support program around the performance of the HEPA differed in its design between years 1 and 2, as summarized in Fig. 1A. At the start of the 2 years, physiotherapists specializing in rheumatology gave instructions and set up training programs tailored to each participant’s abilities. In year 1, physiotherapists tailored training programs to participants’ abilities, offering bi-weekly consultations and 1-h support group meetings (5–10 people) to provide education, feedback, and boost self-efficacy (Nessen et al. 2018). This allowed for participants to share experiences and empower social support, in accordance with the social cognitive theory [16]. In year 2, participants continued the program with optional self-organized support group meetings and handbooks for guidance, without physiotherapist involvement. Full details of the program design are described by Nordgren et al. (2012).

### Self-reporting and anthropomorphic measurements

Participants self-reported weekly the number of performed circuit training sessions and moderate-intensity physical activity for at least 30 min, respectively. Anthropometrical data of weight, waist circumference, and BMI were collected before and after the first and second years of the HEPA intervention.

### Physical fitness assessments

The timed-stands test (TST) assessed lower body functional capacity by measuring the time to sit and stand 10 times with arms crossed [17]. Leg strength, specifically peak torque at 60°/s extension of the quadriceps, was tested with the Biodex System 4 Pro dynamometer. Maximal aerobic capacity was estimated using the Åstrand-Rhyming submaximal bicycle ergometer test, a 6-min bike test based on the linear relationship between heart rate, workload, and maximal capacity (Åstrand and Ryhming, 1954). The hand grip force measurement instrument Grippit was used to measure the maximum momentary force and the average force over a period of 10 s [18]. Trained physical therapists independent of the intervention supervised these tests and collected the data.

### Muscle profiling with mass spectrometry-based proteomics

Muscle tissue (200 µg) was homogenized in 8 M urea, 1 mM dithiothreitol (DTT), and 25 mM HEPES pH 7.6 and processed using 10 kDa filter units (Nanosep, Centrifugal Devices with Omega Membrane 10 k). After centrifugation and alkylation with 25 mM iodoacetamide, proteins were digested overnight at 37 °C with trypsin (1:50 ratio) in a buffer containing 0.25 M urea and 50 mM HEPES. Digested peptides were collected, and concentrations were measured. An internal standard was created by pooling equal peptide amounts from ten samples.

Seventy microgram of protein from each sample was suspended in 0.1 M triethyl ammonium bicarbonate buffer (Sigma-Aldrich) and labelled with Tandem Mass Tag (TMT) 10-plex Isobaric Mass Tagging Kit (ThermoFisher Scientific). Excess reagent was removed using solid phase extraction cartridge (SPE strata-X-C, Phenomenex), and the eluate was dried in a speed-vac. Labelled samples were dissolved in acetonitrile with 0.1% (v/v) formic acid, resulting in a final concentration of 10 µg/µl.

LC–MS/MS was performed on a hybrid Q-Exactive mass spectrometer (ThermoFisher Scientific). Peptides were trapped on an Acclaim PepMap trap column (C18, 3 µm, 100 Å, 75 µm × 20 mm) and separated on an Acclaim PepMap RSLC column (C18, 2 µm, 100 Å, 75 µm × 50 cm) (ThermoFisher Scientific) using a gradient of A (5% dimethyl sulfoxide (DMSO), 0.1% FA) and B (90% ACN, 5% DMSO, 0.1% FA), ranging from 3 to 37% B in 240 min with a flow of 0.25 µl/min. The top ten ions were selected with Fourier transform mass spectrometry (FTMS), and the survey scan was performed at 70,000 resolutions from 400 to 1600 m/z, with a max injection time of 140 ms and a target of 1 × 10^6^ ions. HCD fragmentation spectra were generated at 30% normalized collision energy, 35,000 resolution, and a max ion injection time of 150 ms (AGC target 1 × 10^5^). Precursors were isolated with a width of 2 m/z and put on the exclusion list for 70 s. Single and unassigned charge states were rejected from precursor selection.

MSraw files were analyzed with Proteome Discoverer 1.3 (ThermoFisher Scientific) and the Sequest algorithm against the UniProtKB/SwissProt human database. Data were filtered to 1% FDR, with precursor mass tolerance at 10 ppm and fragment mass tolerance at 0.02 Da. Fixed modifications included carbamidomethylation (C) and TMT (K, N-term), with oxidation (M) as a variable modification. Quantification used only unique peptides, with reporter ions analyzed from HCD-FTMS spectra.

### Data and statistical analysis

Comparisons were made between baseline and year 1, and between baseline and year 2. Differential expression analyses were performed with the DEP R package [19]. The PathfindR bioconductor package was used for enrichment analysis via active subnetworks [20]. Statistical analyses of protein expression between time points were analyzed with GraphPad Prism version 10.0.0 for Windows. Volcano plots were created with the EnhancedVolcano package [21]. Associations between proteins and pathways or functions were calculated with a right-tailed Fisher exact test (*p* < 0.05). Functional test data were analyzed via repeated measures ANOVA.

## Results

### Compliance and changes in body composition with the HEPA intervention

The HEPA intervention was composed of 30 + min of daily moderate-intensity physical activity at least five times per week and 2 × 45-min circuit exercises, which were a combination of strength and aerobic exercise. This resulted in about 300 physically active minutes weekly, of which 255 min and 45 min were allocated to aerobic and muscle-strengthening activities, respectively. Figure 1B–D shows yearly self-reported HEPA sessions, 30-min moderate-intensity aerobic sessions, and circuit training sessions. Participants averaged 259 ± 16 HEPA sessions in year 1, decreasing by 10% in year 2 (Fig. 1B). Moderate-intensity aerobic activity sessions were maintained over the 2 years (Year 1: 207 ± 16; Year 2: 204 ± 16, Fig. 1C), whereas the number of circuit sessions dropped by 45% from year 1 to year 2 (Fig. 1D). Only 7% of the exercise intervention (~ 22 min) per week were allocated to strength training. The intervention implemented moderate effects on body composition with a decreased waist circumference with an average of ~ 2 cm (Fig. 1E) and an average negative body weight change of ~ 2 kg (Fig. 1F), whereas no significant changes in body mass index (BMI) were observed over the 2 years (Fig. 1G).

### Skeletal muscle function improved after year 1 but no further enhancements were observed after 2 years of HEPA

**Fig. 2:**
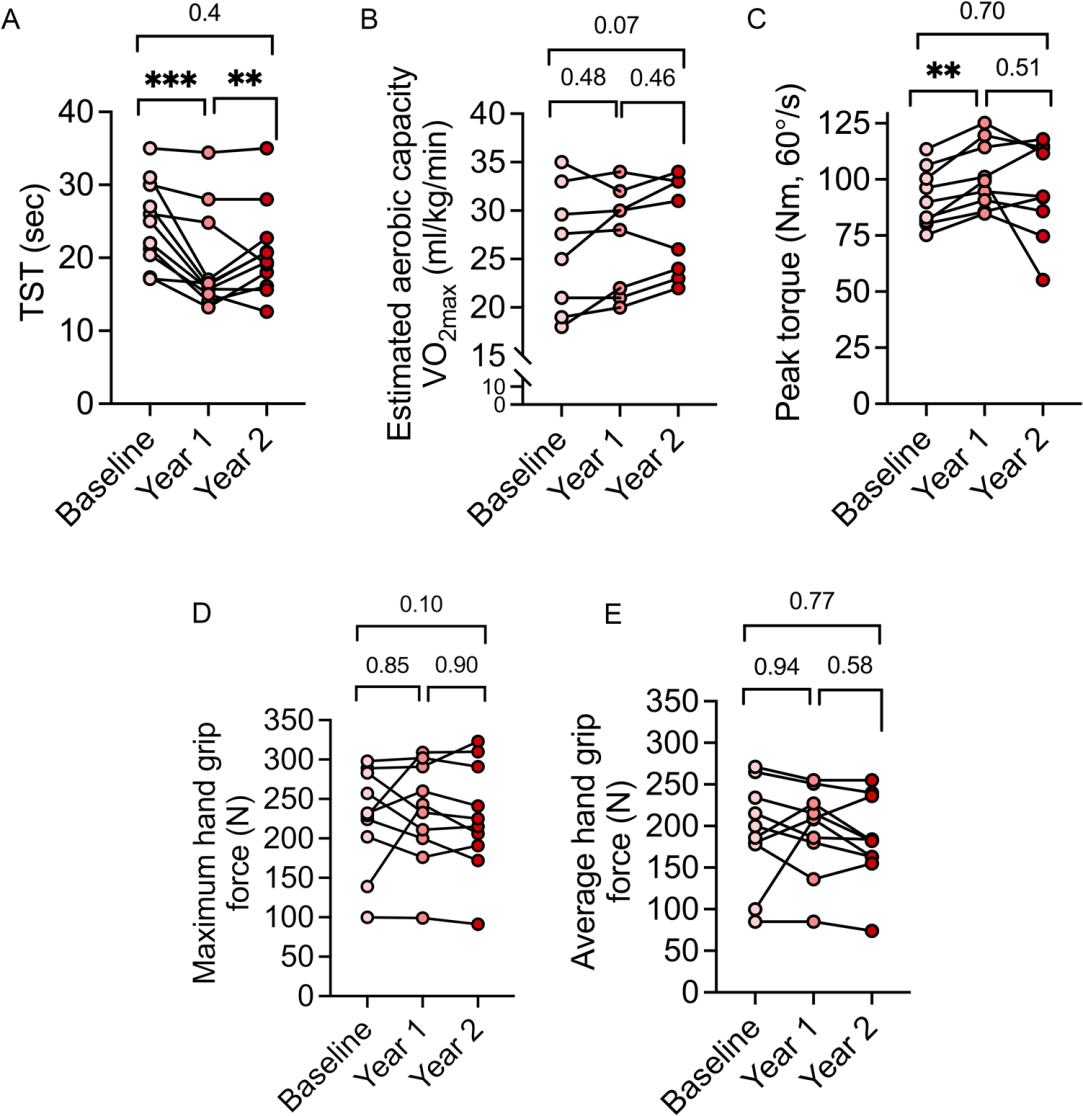
Functional changes induced by HEPA. Graphs displaying functional capacity tests of the subjects throughout the 2 years of the HEPA program; **A** time to stand test (TST), **B** estimated aerobic capacity (VO_2_max, ml/kg/min), **C** peak torque (Nm), and maximum (**D**) and average (**E**) hand grip force in Newton (N). **p < 0.01, repeated measures ANOVA

The average TST time was 24.7 ± 1.7 s at baseline, which improved by ~ 20% after the first year (19.2 ± 1.7 s, *p* = 0.0004), whereas no further improvements were seen after year 2 of HEPA (Fig. 2A). In general, compared with baseline, participants showed an improvement of 4% and 8% after year 1 and year 2, respectively, in the estimated maximal oxygen capacity (VO_2_max) from the Åstrand-Rhyming submaximal ergometer test (Fig. 2B). The peak torque (knee extension 60°/s, average of three measurements per person) produced by quadriceps was 91.8 ± 4.4 Nm at baseline and increased by ~ 10% (101.7 ± 4.9 Nm; *p* = 0.002) after 1 year of training. At the 2-year timepoint, the torque the participants were able to produce had declined by 7% to 95.4 ± 7.1 Nm (Fig. 2C). These data highlight that muscle function improved significantly with HEPA but declined when resistance training sessions were reduced in the second year. In comparison to the first year, only 7% of the total time was allocated to resistance exercise in year 2 (Fig. 1). No HEPA exercises were specifically designed to increase grip strength, and accordingly, no increases in grip strength were observed (Fig. 2D, E). Overall, the improvement in the functional measurements reflects the HEPA design and amount of exercise sessions that were performed.

### Mass spectrometry analyses reveal a HEPA-induced increase followed by a decrease in the expression of mitochondria-associated proteins

**Fig. 3:**
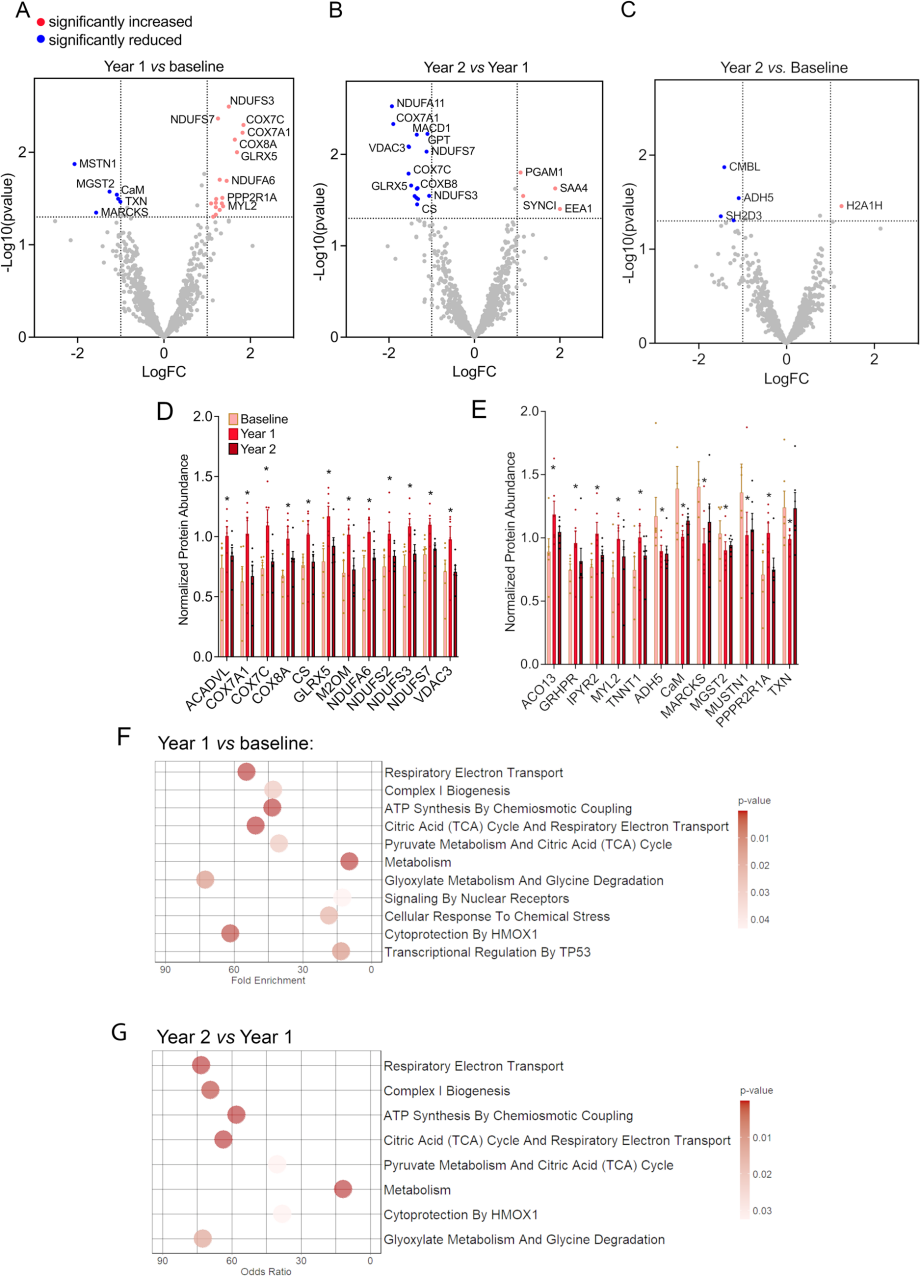
Proteomic analyses of muscle biopsies from HEPA participants. **A**–**C** Volcano plots displaying up and downregulated proteins in **A** year 1 versus baseline, **B** year 1 versus year 2, **C** and year 2 versus baseline. Proteins that are significantly downregulated and upregulated (*p* < 0.05) with a log-fold-change > 1 are highlighted in blue and red, respectively. Bar graphs showing mitochondrial proteins (**D**) and non-mitochondrial proteins (**E**) that were significantly altered after year 1 of the HEPA intervention, and their relation to baseline and year 2, using normalized abundance values from the mass spectrometry analysis. **F**, **G** Gene ontology (GO) enrichment analysis performed using EnrichR comparing year 1 and baseline (**F**) and year 2 and year 1 (**G**)

To elucidate skeletal muscle specific alterations induced by HEPA, vastus lateralis biopsies were collected at baseline and after the first and second years of HEPA. Proteomics identified 961 unique muscle proteins at baseline, with follow-up levels compared to pre-intervention values. Volcano plots illustrated protein expression changes with a fold change ≥ 1 and *p* ≤ 0.05 (Fig. 3A–C). Twenty-five proteins were significantly altered after year 1 of the HEPA program (*p* < 0.05, fold change ≥ 1), of which 18 were increased and seven were reduced (Supplemental Table 2). Of the proteins with higher expression, 67% (12 of 18) were associated with mitochondrial functions (ACADVL, COX7A1, COX7C, COX8A, citrate synthase, GLRX5, M2OM, NDUFA6, NDUFS2, NDUFS3, NDUFS7, and VDAC3), underlining positive aerobic muscle adaptations. The most significantly reduced protein was MUSTN1 (Musculoskeletal embryonic nuclear protein 1, also known as Mustang), which we recently showed to be induced by muscle injury and highly expressed in untrained muscle from subjects with muscle dystrophy by myositis [22].

By the second year, mitochondrial protein expression decreased significantly (Fig. 3B, Supplemental Table 1, *p* < 0.05, fold change ≥ 1), reflecting reduced physical activity in year 2 (Fig. 1). The lack of further adaptation the second year was also evident when comparing biopsies from year 2 with baseline where only five proteins were found to be altered (Fig. 3C, Supplemental Table 4). The abundance of the 25 proteins that were significantly altered after year 1 and their relation to baseline and year 2 are presented in Fig. 3D (mitochondrial proteins) and Fig. 3E (non-mitochondrial proteins). Gene ontology (GO) enrichment analysis performed using EnrichR [23] highlighted enriched pathways linked to mitochondrial biogenesis, including respiratory chain I activity, complex I biogenesis, and ATP synthesis (Fig. 3F, G). By year 2, upregulated proteins had returned to baseline levels, making further enrichment analysis not feasible. Proteomic details are in Supplementary Tables 2–4.

**Fig. 4:**
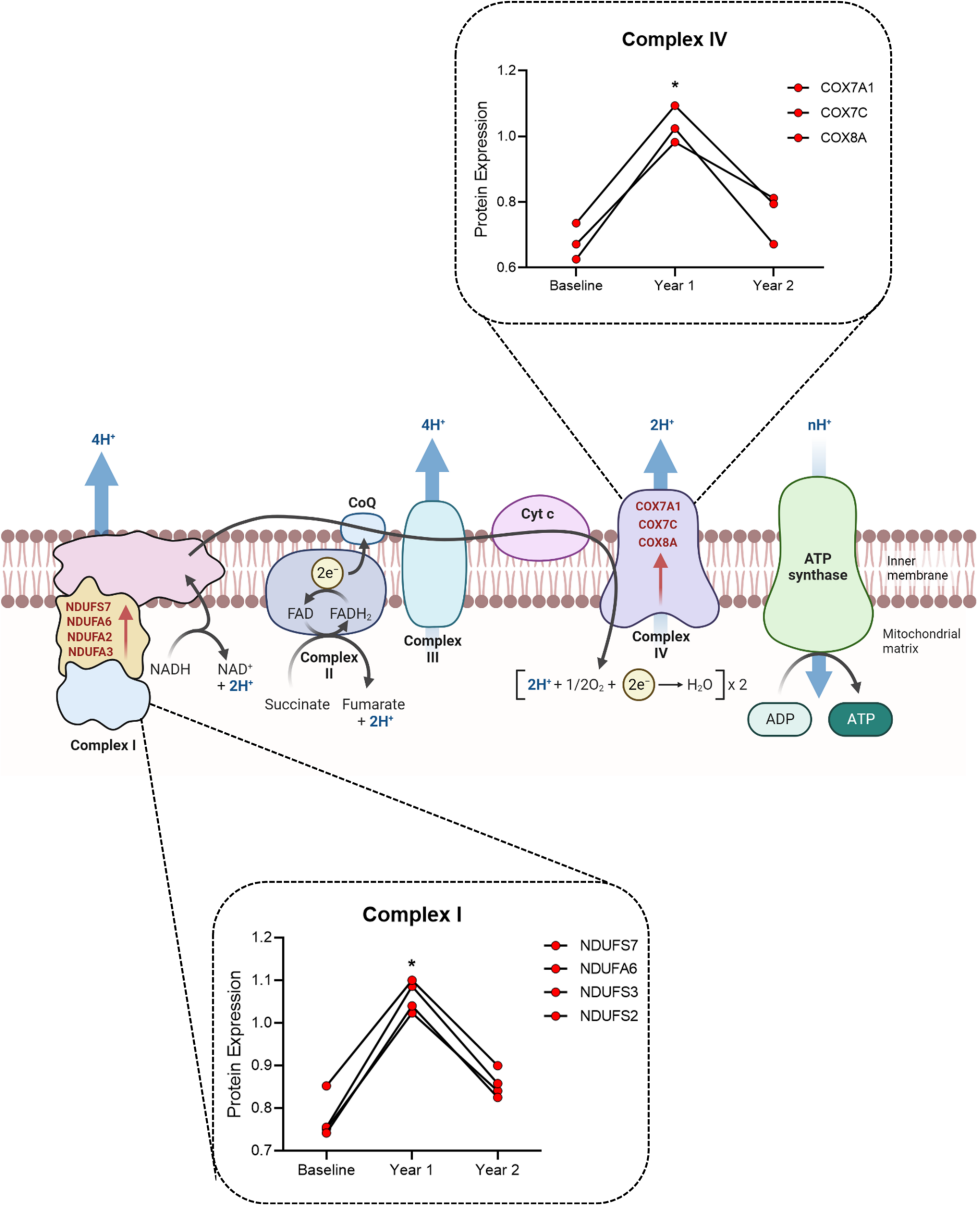
The electron transport chain and associated mitochondrial complexes. Graphs indicate proteins that were significantly differentially expressed in mitochondrial complex I and IV, as determined by mass spectrometry. Comparisons of protein expression in the different time points were analyzed via repeated measures ANOVA. *p < 0.05. Image created in BioRender

Four of the enriched proteins identified after year 1 of the HEPA intervention are associated with complex I of the mitochondria respiratory chain: NDUFS7, NDUFA2, NDUFA3, and NDUFA6 (Fig. 4). Moreover, three proteins that belong to complex IV (cytochrome C oxidase) were also significantly upregulated: COX7A1, COX7C, and COX8A (Fig. 4). As illustrated in Fig. 4, the changes that occurred after the first year returned to baseline after year 2; hence, no further mitochondrial adaptation occurred during the second year, which corresponded with a reduction in the exercise frequency.

## Discussion

This study demonstrates for the first time that HEPA can induce beneficial mitochondrial adaptations in the skeletal muscle proteome of individuals with RA. However, these adaptations declined when exercise was not maintained in the second year, aligning with findings from healthy individuals [24]. The results highlight the critical role of sustained support and motivation strategies in maintaining adherence and achieving long-term exercise benefits. Building on these findings, we propose that the ideal exercise program for RA patients should combine aerobic and resistance training, with more weekly exercise time dedicated to resistance exercises to maintain muscle function and enhance mitochondrial adaptations. The program should progressively increase in intensity and duration, be tailored to individual capabilities, and incorporate regular physiotherapy or group sessions to foster accountability and motivation. Flexibility in timing and modalities, such as home-based workouts or digital coaching, can address logistical barriers, while progress assessments using fitness tests or wearable trackers can help sustain motivation and adherence.

Pathway analyses revealed significant enrichment of mitochondria-associated pathways after the first year of the intervention, with 12 of 18 upregulated proteins linked to mitochondrial functions. Exercise is known to enhance mitochondrial content and function by increasing key regulators of mitochondrial biogenesis, such as PGC-1, NRF1, and NRF2 [25, 26]. This has not only been observed in healthy individuals [27–29] but also in patients with chronic conditions such as type 2 diabetes [30] and cardiovascular disease [31].

Mitochondrial function in the skeletal muscles of individuals with RA is poorly understood, but recent findings indicate reduced mitochondrial respiratory capacity and content compared to healthy controls [32]. Our study demonstrates that 1 year of HEPA significantly enhanced mitochondrial health in individuals with RA. Complexes I and IV of the respiratory chain were notably enhanced, with increased expression of subunits such as NDUFS7, NDUFA6, NDUFA2, NDUFA3, COX7A1, COX7C, and COX8A. Additionally, citrate synthase (CS) and acetyl-CoA dehydrogenase (ACADVL), key to the TCA cycle and fatty acid oxidation, also increased. These results align with studies in aging populations, where structured exercise improved complex I activity and lipid metabolism [33].

Elevated levels of mitochondrial proteins are linked to enhanced aerobic capacity and skeletal muscle fitness. In our study, the TST, which assesses lower extremity skeletal muscle function, increased by 20% after the first year, with no further increases in the second year. The bicycle test to assess VO_2_max also showed a positive trend in the first year but did not reach statistical significance as not all individuals improved. This discrepancy is partly expected and explained by TST being simpler, focusing on thigh muscle fitness, whereas the VO2max test assesses whole-body aerobic capacity and requires cycling proficiency. Reduced cardiorespiratory fitness is associated with an increased risk of cardiac disease, and previous research has indicated that individuals with RA typically have 20 to 30% lower aerobic capacity than age-matched healthy controls [34]. However, most participants in this cohort did not exhibit such drastic reductions. Additionally, the trainability of VO2max has been shown to be relatively limited. Thus, the effects elicited by this exercise intervention may be more difficult to detect [35]. Furthermore, it is unclear how many HEPA sessions were performed at intensity levels that would result in measurable increases in aerobic capacity.

As well as the changes in mitochondrial proteins, this study also identified a significant downregulation of MGST2 after the first year of HEPA, which may be a novel marker of skeletal muscle health. In general, little is known about MGST2, but Dvash and colleagues have shown that MGST2 activation contributes to the accumulation of excess reactive oxygen species (ROS) [36], a process we have previously shown to contribute to muscle weakness in RA [3]. Moreover, MGST2 reduction by silencing using siRNA was shown to abolish the aberrant ROS and protect against DNA damage in vitro and in mouse kidneys [36]. Future studies to further explore the role of MGST2 in skeletal muscle and how exercise influences its activity are warranted.

Calmodulin (CaM), a well-known Ca^2+^ binding protein, was downregulated after 1 year of the HEPA intervention. CaM activates Ca^2+^/calmodulin-dependent kinases (CaMKs), which regulate Ca^2^ + handling, myosin type IIA expression, and mitochondrial biogenesis [37, 38]. CaM also regulates genes central to T-cell proliferation and activation [39]. In RA, altered metabolic phenotypes of naïve CD4 + T cells contribute to inflammation [40]. Indeed, the effects of Ca^2+^/CaM signaling on the immune and inflammatory responses have been extensively explored [41–43], and inhibitors of CaM signaling (such as calcineurin) are used in the clinic for the prevention of inflammatory diseases, including RA [44, 45]. In skeletal muscle, research found CaM-dependent signaling pathways to actively mediate the acute inflammatory response [46]. The observed reduction in CaM may indicate a shift toward an anti-inflammatory muscle phenotype, aligning with reduced adherence to the HEPA intervention in year 2, when CaM levels returned to baseline.

Except for slow skeletal muscle troponin (TNNT1), there were no myofibrillar proteins enriched at any time point in the proteomics analyses, which reflects the lack of resistance training included in HEPA (only 15% of HEPA was allocated to resistance training). This lack of hypertrophy was corroborated by a loss of lean mass over 2 years (baseline vs. year 1: − 0.89 ± 0.37 kg, *p* = 0.03 and baseline vs. year 2: − 0.08 ± 0.24 kg, *p* = 0.96. *N* = 15). Despite this, torque improved by ~ 10% after the first year, likely driven by enhanced muscle function rather than increased myofibrillar content. Previous work by [3] demonstrated impaired specific force in individuals with RA, suggesting that functional gains from HEPA may compensate for intrinsic deficits in muscle quality.

This study demonstrated beneficial skeletal muscle adaptations in individuals with RA following a health-enhancing physical activity (HEPA) intervention. However, the study is not without limitations. One notable constraint is the small cohort size, which reflects the exploratory nature of the study. Unlike the larger parent studies by Nordgren et al. [9, 11] that determined robust sample size requirements and demonstrated significant outcomes in 177 participants over 2 years, our study focuses on mechanistic insights into skeletal muscle adaptations induced by HEPA. By leveraging paired biopsies and proteomic analysis, we aimed to generate hypotheses and identify pathways underlying these benefits, providing a basis for future confirmatory studies. Additionally, while our study included only female participants, consistent with RA’s higher prevalence in women, we recognize that this limits the generalizability of our findings to male patients. Moreover, while the data from repeated biopsies confirmed HEPA’s positive effects on mitochondrial function, only a small number of proteins met the thresholds for fold-change and statistical significance. Future studies should leverage more advanced proteomic technologies to enhance the biological relevance of findings. Additionally, simultaneous analysis of skeletal muscle and blood could provide deeper insights into the systemic benefits of exercise.

In conclusion, this study shows that long-term exercise, like HEPA, improves physical capacity and mitochondrial function in individuals with RA. However, these benefits depend on adherence, which dropped in the second year when physiotherapy and support group meetings were reduced. This highlights the importance of accountability in long-term exercise programs. While HEPA enhances aerobic capacity and mitochondrial proteins, sustaining and progressing the exercise regimen is crucial to maintaining these beneficial adaptations.

## Supplementary Information

Below is the link to the electronic supplementary material.

Supplementary file1 (XLSX 26 KB)

## Data Availability

Data generated and analysed during this study are available from the corresponding author upon reasonable request.

## Abbreviations

*ACADVL*: Acetyl-CoA dehydrogenase
*ACR*: American College of Rheumatology
*ATP*: Adenosine triphosphate
*ADP*: Adenosine diphosphate
*CS*: Citrate synthase
*DAS 28*: Disease Activity Score 28 joints
*FTMS*: Fourier transform mass spectrometry
*DTT*: Dithiothreitol
*HEPA *: Health-enhancing physical activity
*LC–MS/MS*: Liquid chromatography mass spectrometry MS
*RA*: Rheumatoid arthritis
*RM *: Repetition maximum
*SCT *: Social cognitive theory
*SRQ*: Swedish Rheumatology Quality register
*TST*: Time stands test
*TTM *: Transtheoretical model
*TMT*: Tandem Mass Tag
*WHO*: World Health Organization
*COX7A1*: Cytochrome c oxidase subunit 7A1
*COX7C*: Cytochrome c oxidase subunit 7C
*COX8A*: Cytochrome c oxidase subunit 8A
*CS*: Citrate synthase
*GLRX5*: Glutaredoxin 5
*M2OM*: Mitochondrial 2-oxoglutarate
*NDUFA6*: NADH dehydrogenase [ubiquinone] 1 alpha subcomplex subunit 6
*NDUFS2*: NADH dehydrogenase [ubiquinone] iron-sulfur protein 2
*NDUFS3*: NADH dehydrogenase [ubiquinone] iron-sulfur protein 3
*NDUFS7*: NADH dehydrogenase [ubiquinone] iron-sulfur protein 7
*VDAC3*: Voltage-dependent anion channel 3
*MUSTN*: Musculoskeletal embryonic nuclear protein 1
*CaM*: Calmodulin
*PGC-1a*: Peroxisome proliferator-activated receptor gamma coactivator 1-alpha
*NRF1*: Nuclear respiratory factor 1
*NRF2*: Nuclear respiratory factor 2
*MGST2*: Microsomal glutathione S-transferase 2
*TNNT1*: Slow skeletal muscle troponin T
